# Clinical features and treatment challenges of HER2-positive primary breast squamous cell carcinoma: a case report and literature review

**DOI:** 10.3389/fonc.2026.1781401

**Published:** 2026-07-09

**Authors:** Fang Yang, Siyu Guo, Ping Li, Mengqi Yang, Xuan Wu

**Affiliations:** 1 Department of Oncology, Peking University Shenzhen Hospital, Shenzhen, China; 2 Department of Radiotherapy, Peking University Shenzhen Hospital, The First School of Clinical Medicine, Guangdong Medical University, Shenzhen, China; 3 Department of Pathology, Peking University Shenzhen Hospital, Shenzhen, China; 4 Department of Radiotherapy, Peking University Shenzhen Hospital, Shenzhen, China; 5 Department of Oncology, Peking University Shenzhen Hospital, The First School of Clinical Medicine, Guangdong Medical University, Shenzhen, China

**Keywords:** antigen loss, breast squamous cell carcinoma, HER2 amplification, *PIK3CA* mutation, precision therapy

## Abstract

Primary breast squamous cell carcinoma (PBSCC) with HER2-positive status is exceptionally rare, with fewer than 100 cases reported globally, and HER2 positivity occurring in only 5.8-7.1% of these cases. No established treatment standards exist for this entity. We present the case of a 43-year-old woman with HER2-positive PBSCC who exhibited a poor response to neoadjuvant TCHP therapy (Miller-Payne grade 2). Local recurrence occurred just 3 months after mastectomy. Second-line therapy with pyrotinib plus capecitabine provided 9 months of disease control before lung metastasis emerged. Subsequent molecular profiling revealed a *PIK3CA* E545K mutation (VAF 40.3%), co-amplified with *FGF3/4/19* and *CCND1*. Although third-line treatment with trastuzumab deruxtecan (T-DXd) achieved a partial response, the progression-free survival (PFS) was limited to only 5 months. A repeat biopsy confirmed HER2 downregulation (from 3+ to 2+), identifying antigen loss as a key mechanism of acquired resistance. A review of the literature indicates that the pathological complete response rate of HER2-positive PBSCC to standard HER2-targeted therapy is remarkably low, far inferior to the 50-60% pCR rates achieved with dual HER2 blockade in HER2-positive invasive ductal carcinoma. This case underscores that upon failure of HER2-targeted therapy accompanied by HER2 antigen loss, the treatment strategy should pivot towards molecularly-guided precision therapy. Based on evidence such as that from the TRIUMPH trial, priority should be given to agents targeting the detected alterations, such as *PIK3CA* or *FGFR* inhibitors, rather than persisting with HER2-targeted approaches.

## Graphical Abstract

**Figure:**
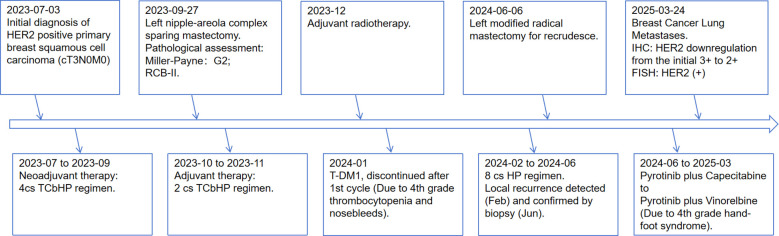

## 1 Introduction

### 1.1 Extreme rarity of the disease

HER2-positive primary breast squamous cell carcinoma (PBSCC) represents a “rare within rare” malignancy. PBSCC itself constitutes less than 0.1% of all breast carcinomas (1, 2), and HER2 overexpression within this subtype is distinctly uncommon. Consequently, the actual incidence of HER2-positive PBSCC is estimated at a mere 0.006-0.007%. With fewer than 100 confirmed cases documented worldwide, as evidenced by cumulative case series and literature reviews (1–4), it ranks among the scarcest molecular subtypes in breast oncology.

This extreme rarity poses significant clinical challenges, including a lack of standardized treatment guidelines, difficulties in clinical trial enrollment, and a severe scarcity of prognostic and therapeutic response data. Current clinical experience is predominantly derived from isolated case reports. Therefore, each comprehensively documented case holds exceptional value for guiding future practice.

### 1.2 Complex therapeutic challenges in HER2-positive PBSCC

As a distinct subtype of metaplastic breast cancer, primary breast squamous cell carcinoma (PBSCC) presents predominantly (in ~89.7% of cases) as triple-negative breast cancer (4, 5). This prevalence underscores the unique clinical context of HER2-positive PBSCC, a rare variant that demands therapeutic strategies divergent from those used for conventional HER2-positive invasive ductal carcinoma (IDC). Clinically, this distinction is critical: while dual HER2-targeted neoadjuvant therapy typically achieves pathological complete response (pCR) rates of 50–60% in HER2-positive IDC (6, 7), the corresponding pCR rate reported for HER2-positive PBSCC is only 12.5% (1 of 8 cases) in the largest available case series to date (8), highlighting its inherent resistance to standard HER2-directed regimens. The aggressive behavior and treatment resistance observed in PBSCC are increasingly attributed to its distinct biology. Key mechanisms under investigation include constitutive activation of epithelial-mesenchymal transition (EMT) programs (5, 9), frequent alterations driving PI3K/AKT pathway hyperactivation (10, 11), and features of a complex tumor microenvironment that may limit drug delivery and efficacy (12, 13). In the setting of metastatic HER2-positive breast cancer, the antibody-drug conjugate (ADC) trastuzumab deruxtecan (T-DXd) has established a new standard of care, based on the landmark DESTINY-Breast03 trial (14). However, its efficacy specifically in rare, biologically distinct subtypes such as HER2-positive PBSCC remains almost entirely uncharacterized, as these patients are invariably excluded from large-scale clinical trials. This case report aims to address this evidence gap. We provide a comprehensive longitudinal analysis of a patient with HER2-positive PBSCC, tracing the molecular evolution of the disease across multiple lines of therapy until ultimate progression. Our objective is to illuminate the clinical decision-making challenges posed by this malignancy and to offer actionable insights for managing therapeutic resistance.

## 2 Case report

### 2.1 Initial diagnosis and neoadjuvant therapy

In July 2023, a 43-year-old premenopausal woman presented with a self-detected left breast mass. Physical examination revealed a 5.0 × 5.0 cm irregular, firm mass with limited mobility. Mammography and ultrasound identified a 5.0 × 4.9 × 4.3 cm BI-RADS category 5 mass with microcalcifications (**Figure 1**). Core needle biopsy established the diagnosis of invasive carcinoma with squamous differentiation (Grade 3). Immunohistochemical analysis demonstrated HER2 3+ positivity, weak estrogen receptor expression (2%), negative progesterone receptor, and a Ki-67 proliferation index of 70%. Fluorescence *in situ* hybridization confirmed HER2 gene amplification (15 copies). Representative HE and immunohistochemical images (Ki-67, HER2, ER) are shown in **Figure 2**. Staging workup established the diagnosis as cT3N0M0 (Stage IIA) disease.

**Figure 1:**
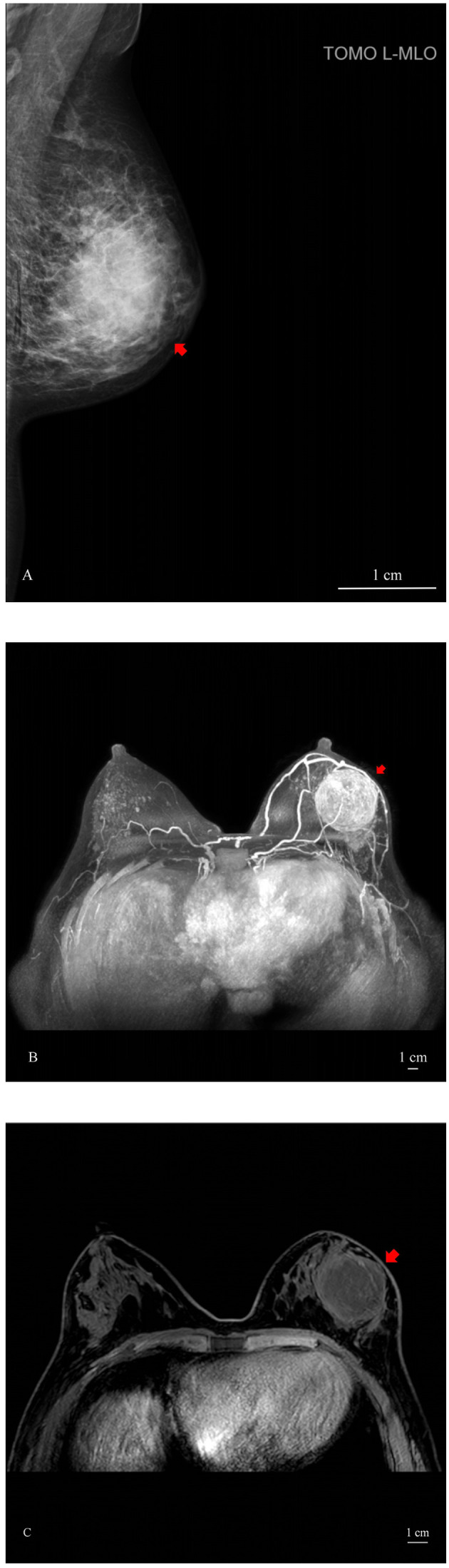
Imaging data at initial diagnosis before treatment. **(A)** Digital breast tomosynthesis (TOMO, 2023-07-05): A mass measuring approximately 55×45×60 mm with microcalcifications in the left breast, classified as BI-RADS 5. **(B, C)** Breast magnetic resonance imaging (MRI, 2023-07-13): An irregular, rim-enhancing mass measuring approximately 50×49×43 mm in the left breast with restricted diffusion, classified as BI-RADS 5.

**Figure 2:**
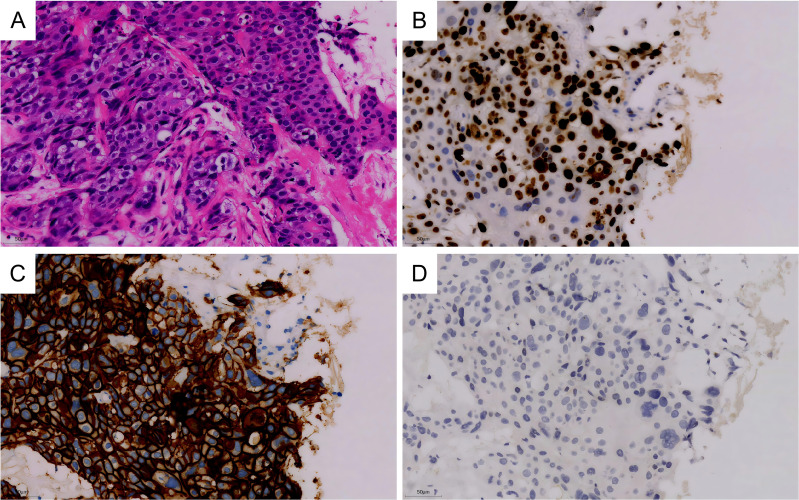
Histopathology and immunohistochemistry of the initial core needle biopsy (×40). **(A)** Hematoxylin and eosin (HE) staining showing invasive carcinoma with squamous differentiation. **(B)** Ki-67 immunostaining, with a proliferation index of approximately 70%. **(C)** HER2 immunostaining showing strong, complete membranous reactivity (3+). **(D)** Estrogen receptor (ER) immunostaining showing weak expression (approximately 2%).

Between July and September 2023, the patient received four cycles of neoadjuvant therapy with the TCHP regimen (docetaxel, carboplatin, trastuzumab, and pertuzumab). Treatment response was evaluated as inadequate. Post-therapy magnetic resonance imaging revealed minimal tumor reduction from 5.4 cm to 4.6 cm, meeting RECIST 1.1 criteria for stable disease.

### 2.2 Surgical intervention and pathological assessment

On September 27, 2023, the patient underwent left nipple-areola complex-sparing mastectomy with sentinel lymph node biopsy, axillary lymph node dissection, and prosthesis implantation. Histopathological examination of the surgical specimen confirmed metaplastic carcinoma with predominant squamous cell differentiation (moderately differentiated) (**Figure 3**). The tumor bed evaluation showed a Miller-Payne grade 2 response with 85% residual tumor cellularity, corresponding to Residual Cancer Burden class II (RCB-II). Lymph node examination revealed no metastatic involvement (0/6 nodes). Representative sections from three of the sampled nodes are shown in **Figure 4**. Lymphovascular invasion and perineural invasion were identified.

**Figure 3:**
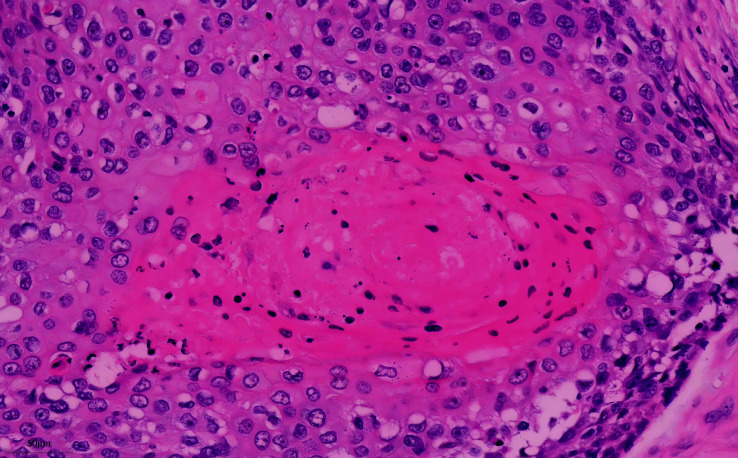
HE staining of the surgical specimen (×40). Demonstrating metaplastic carcinoma with predominant squamous differentiation.

**Figure 4:**
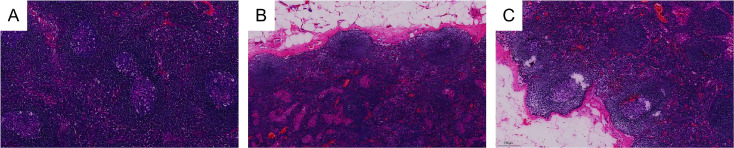
HE staining of three sampled lymph nodes (×10). **(A–C)** Representative sections of three separate lymph nodes which are all negative for metastatic involvement (0/6).

### 2.3 Adjuvant therapy and first recurrence

The postoperative treatment course included two cycles of TCbHP regimen chemotherapy between October and November 2023, followed by adjuvant radiotherapy in December 2023. In January 2024, treatment with T-DM1 was initiated but discontinued after the first cycle due to grade 4 thrombocytopenia complicated by severe epistaxis requiring emergency intervention. From February to June 2024, the patient completed eight cycles of dual HER2-blockade with trastuzumab and pertuzumab.

In February 2024, a nodule was detected at the mastectomy scar site, which progressively enlarged with associated cutaneous erythema and tenderness. Pathological examination of a biopsy specimen obtained on June 3, 2024, confirmed malignant recurrence. The patient subsequently underwent left modified radical mastectomy on June 6, 2024 (**Figure 5**). Surgical pathology confirmed recurrent metaplastic carcinoma with predominant squamous cell components, maintaining HER2 3+ expression, Ki-67 of 70%, and weak ER expression (2%).

**Figure 5:**
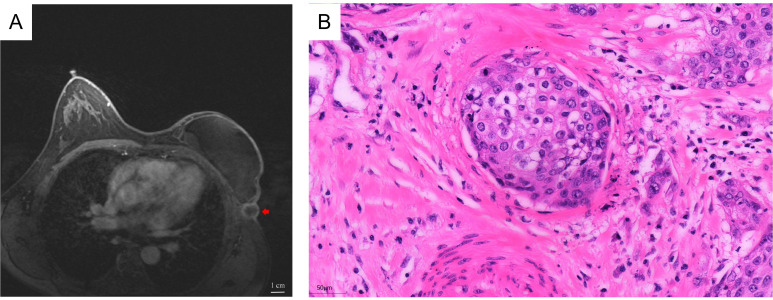
First local recurrence on the chest wall. **(A)** Breast MRI (2024-06-05): a well-defined, round nodule (approximately 17×17×18 mm) in the subcutaneous tissue of the left chest wall, suggestive of local recurrence. **(B)** HE staining of the recurrent surgical specimen (2024-06-06, ×40), confirming recurrent metaplastic carcinoma with predominant squamous components.

### 2.4 Management of recurrent disease and progression

Following surgery, the patient initiated second-line therapy with pyrotinib plus capecitabine. In December 2024, capecitabine was switched to vinorelbine (with pyrotinib maintained) due to grade 4 hand-foot syndrome. Surveillance computed tomography of the thorax performed on March 3, 2025, demonstrated interval enlargement of a solid nodule in the left lower lobe, consistent with metastatic progression, along with new pulmonary nodules in the right lung (**Figure 6**).A subsequent CT-guided needle biopsy of the left lower lobe lesion on March 24, 2025, confirmed metastatic disease. Third-line therapy with trastuzumab deruxtecan (T-DXd) was subsequently initiated, achieving a partial response; however, progression-free survival was limited to 5 months before further progression.

**Figure 6:**
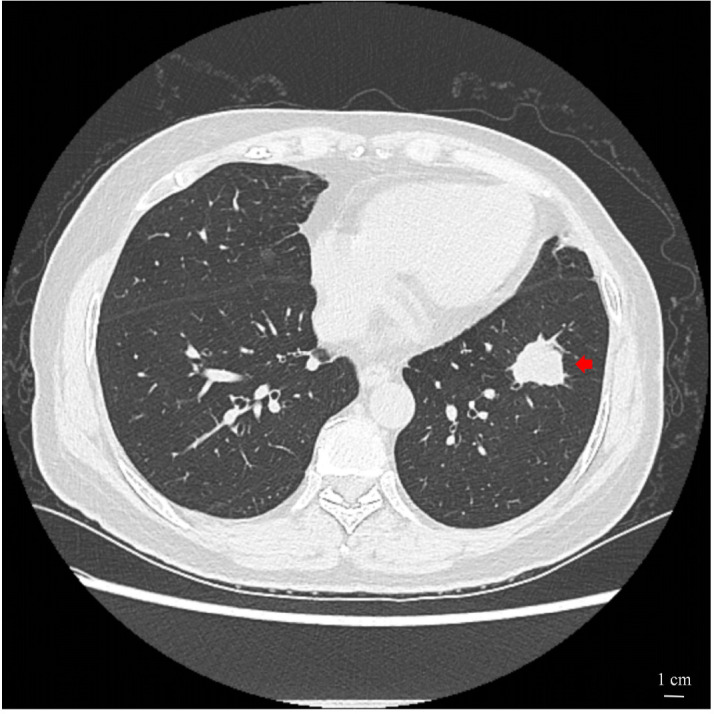
Newly developed pulmonary lesion. Chest CT (2025-03-24): a solid nodule (approximately 26×21 mm) in the left lower lobe, raising concern for metastatic disease.

### 2.5 Molecular profiling and resistance mechanisms

Comprehensive molecular analysis of archived primary tumor tissue identified a *PIK3CA* E545K mutation (variant allele frequency 40.3%) with concurrent amplification of *FGF3/4/19* and *CCND1*, indicating simultaneous activation of PI3K and *FGFR* signaling pathways. Biopsy of a progressing pulmonary metastatic lesion revealed HER2 downregulation from the initial 3+ to 2+ by immunohistochemistry (IHC), while fluorescence *in situ* hybridization (FISH) maintained positivity for HER2 gene amplification (**Figure 7**). This gene-protein discordance established HER2 antigen loss as the principal mechanism of acquired resistance to trastuzumab deruxtecan.

**Figure 7:**
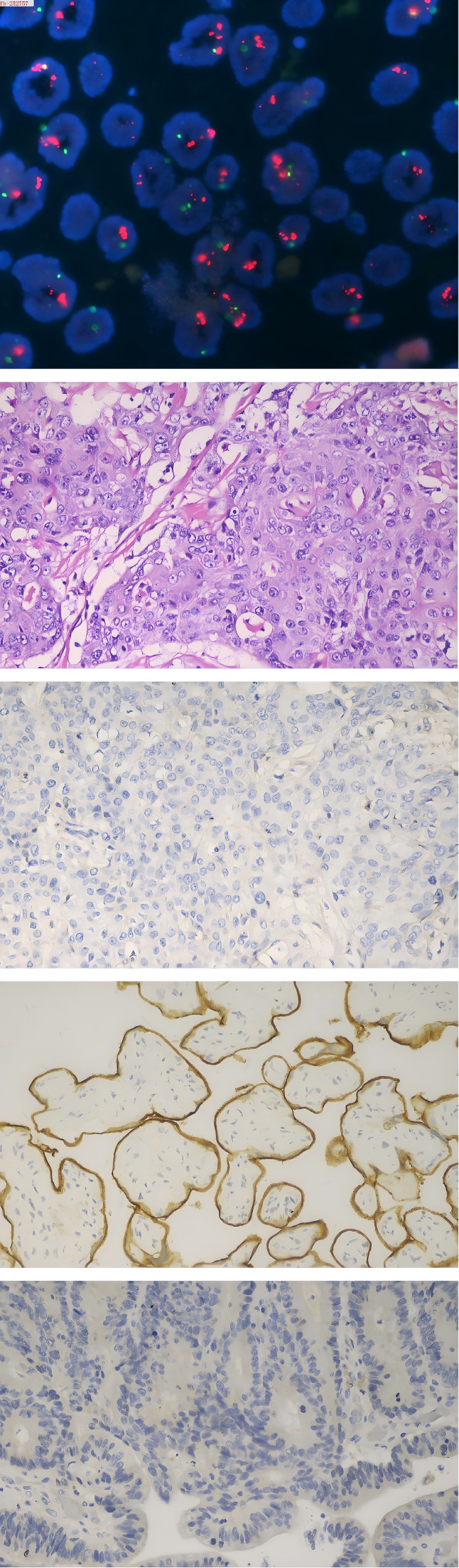
Biopsy of the pulmonary metastatic lesion (2025-03-24). HER2 protein expression is downregulated to 2+ by IHC, while FISH confirms persistent HER2 gene amplification.

## 3 Mechanisms of treatment resistance

### 3.1 Basis for primary resistance to neoadjuvant therapy

The profound resistance to the TCHP regimen observed in this case can be attributed to three synergistic factors. First, the characteristic histology of squamous cell carcinoma, featuring abundant stroma and relatively poor vascularity, creates a “physical barrier” that likely impedes the effective delivery of both chemotherapeutic and antibody-based drugs—a microenvironmental feature particularly prominent in PBSCC (9, 13).

In this patient, next-generation sequencing of the archival primary tumor identified a high-clonality *PIK3CA* E545K mutation at a variant allele frequency (VAF) of 40.3%. Given the tumor purity, this VAF approaches the fraction expected for a heterozygous truncal mutation, indicating that the alteration was an early clonal event rather than a late subclonal acquisition—consistent with its presence at diagnosis and its likely contribution to primary TCHP resistance. This helical-domain mutation drives ligand-independent PI3K/AKT/mTOR activation (15, 16), providing a HER2-independent survival signal; *PIK3CA* mutations have been associated with reduced benefit from neoadjuvant HER2-targeted therapy (17).

Sequencing of the same specimen demonstrated concurrent co-amplification of *FGF3/4/19* and *CCND1*. The temporal correlation in this case is informative: the tumor failed, in rapid succession, neoadjuvant TCHP, adjuvant T-DM1 and trastuzumab–pertuzumab, and second-line pyrotinib—a sequential collapse of HER2-directed regimens that mirrors the multi-driver genotype detected at baseline. Mechanistically, *FGFR* pathway activation is a recognized mediator of resistance to HER2-directed therapy (18, 19), while *CCND1* amplification accelerates the cell cycle through CDK4/6 (20). Together, these patient-specific alterations defined a bypass network operating independently of HER2 signaling.

### 3.2 Mechanism of acquired resistance to trastuzumab deruxtecan

The CT-guided needle biopsy of the lung metastasis provided direct clinicopathological evidence for the mechanism of acquired resistance to T-DXd: the downregulation of HER2 protein expression from IHC 3+ to 2+ (**Figure 7**). Notably, FISH analysis confirmed persistent HER2 gene amplification despite reduced protein expression, establishing a classic gene-protein discordance characteristic of antigen loss. To evade the cytotoxic payload of T-DXd, tumor cells reduced HER2 surface density, thereby impairing the binding, internalization, and efficacy of the antibody-drug conjugate (21, 22).

Under the selective pressure of T-DXd treatment, tumor subclones capable of downregulating HER2 expression while sustaining proliferation through alternative pathways (e.g., PI3K or *FGFR*) were positively selected. This clonal evolution explains the transient partial response to T-DXd (progression-free survival of 5 months)—achieved through its potent topoisomerase I inhibitor payload—and the ultimate treatment failure as HER2-low, bypass-activated resistant clones emerged as the dominant population. Thus, in this patient the 5-month PFS on T-DXd, the documented IHC 3+→2+ shift with retained FISH amplification, and the pre-existing PI3K/*FGFR* bypass network together constitute a single, internally consistent chain of evidence linking clonal evolution to treatment failure—observed directly in this case rather than inferred from external series.

## 4 Discussion

### 4.1 A convergent model of therapeutic resistance in HER2-positive PBSCC

The clinical trajectory of our patient with HER2-positive PBSCC reveals a consistent pattern of therapeutic resistance that extends beyond the typical mechanisms observed in conventional HER2-positive breast cancer. This case provides clinical evidence for a multi-layered resistance model, where histological, molecular, and evolutionary barriers converge to undermine HER2-targeted strategies.

The first barrier appears to be histological. The characteristically dense stroma and compromised vasculature of squamous cell carcinomas likely create a physical impediment to drug delivery (9, 13). This microenvironmental feature may explain the limited efficacy of both chemotherapeutic and antibody-based agents in our case, where the tumor showed only minimal regression following neoadjuvant TCHP despite HER2 overexpression.

At the molecular level, our case demonstrates how concurrent genomic alterations create robust bypass signaling networks. The presence of a high-VAF *PIK3CA* E545K mutation alongside *FGF3/4/19* and *CCND1* co-amplification suggests that HER2 amplification exists within a multi-driver context rather than as a solitary oncogenic dependency. The *PIK3CA* mutation sustains PI3K/AKT/mTOR signaling independent of HER2 inhibition (15, 16), while *FGFR* amplification provides an alternative receptor tyrosine kinase pathway (18, 19), and *CCND1* amplification directly accelerates cell cycle progression (20). This molecular constellation creates multiple escape routes from HER2-directed therapy.

Most significantly, the acquisition of HER2 antigen loss under T-DXd selective pressure represents the ultimate evolutionary adaptation. The documented reduction from HER2 3+ to 2+ expression despite persistent gene amplification demonstrates how these tumors can dynamically eliminate the very target of therapy (21, 22). This finding has profound clinical implications, as it fundamentally redefines HER2 positivity in PBSCC as a potentially transient state rather than a stable therapeutic vulnerability.

### 4.2 Contextualizing our findings within the PBSCC literature

The resistance patterns observed in our patient align with the consistently poor outcomes reported for HER2-positive PBSCC across the literature. Systematic reviews indicate that fewer than 100 cases have been documented worldwide (1–4), with HER2 positivity present in only 5.8-7.1% of all PBSCC cases (3, 4). These tumors typically present as large, hormone receptor-negative masses with high proliferation indices, predominantly affecting middle-aged to elderly women (2, 8).

The molecular landscape of PBSCC provides important context for our findings. Whole-exome sequencing studies have revealed a genomic profile distinct from conventional breast cancer, with high frequencies of *TP53* (60-75%), *PIK3CA* (30-40%), and various cell cycle regulators (5, 10, 11). This molecular signature more closely resembles that of head and neck squamous carcinomas than typical breast malignancies, suggesting shared oncogenic mechanisms (23–25).

Treatment outcomes for HER2-positive PBSCC remain dismal across reported cases. The pCR rates with neoadjuvant HER2-targeted therapy are remarkably low (8, 26), far below the 50-60% rates observed with dual HER2 blockade in HER2-positive IDC (6, 7). In the metastatic setting, median PFS is typically 3–8 months (8), and experience with novel agents like T-DXd remains limited to isolated case reports (14, 22). Our patient’s 5-month PFS on T-DXd aligns with this pattern, though our documentation of antigen loss provides mechanistic insight previously unavailable in the literature.

### 4.3 The biological divergence between PBSCC and conventional HER2-positive breast cancer

The profound disparity in treatment outcomes between HER2-positive PBSCC and IDC reflects a fundamental biological distinction. While IDC often demonstrates “oncogene addiction” to HER2, making it highly vulnerable to pathway blockade, PBSCC appears to function as a “multi-driver” disease from its inception. The squamous cell lineage of PBSCC predisposes it to a genomic landscape rich in concomitant *PIK3CA*, *FGFR*, and cell cycle alterations, which cooperate with HER2 but do not create exclusive dependency on it.

This biological difference explains why therapeutic strategies optimized for HER2-addicted IDC prove fundamentally mismatched against the resilient, multi-pathway architecture of PBSCC. This is exemplified by the dramatically lower pCR rates with dual HER2 blockade in HER2-positive PBSCC (12.5%) (8) compared to HER2-positive IDC (50–60%) (6, 7), underscoring the need for biology-informed approaches. The convergence of alternative signaling pathways in PBSCC creates a biological buffer against HER2 inhibition, while the histopathological features further limit drug efficacy. Our case demonstrates how this inherent resistance can culminate in complete target elimination through antigen loss, representing the final step in evolutionary escape from HER2-directed therapy.

### 4.4 Clinical implications: toward a biology-informed treatment paradigm

Our findings, contextualized within the existing literature, suggest the need for a paradigm shift in managing HER2-positive PBSCC. Rather than persisting with sequential HER2-targeted regimens, a biology-informed approach should guide therapeutic decisions.

First, baseline comprehensive molecular profiling appears essential for treatment planning in HER2-positive PBSCC. Given the high frequency of co-alterations, the presence of concurrent *PIK3CA* mutations or *FGFR* amplifications may warrant consideration of combination strategies or earlier transition to alteration-specific therapies.

Second, repeat biopsy at progression emerges as a critical diagnostic procedure, particularly following ADC therapy. Our case demonstrates that antigen loss can fundamentally alter the therapeutic landscape, rendering continued HER2-directed approaches ineffective. Documentation of such resistance mechanisms should guide subsequent treatment selection.

Third, upon confirmation of HER2 antigen loss, therapeutic focus should shift decisively toward targeting the underlying squamous drivers. The *PIK3CA* E545K mutation identifies a population that may derive particular benefit from PI3Kα inhibitors, as suggested by trials in *PIK3CA*-mutant malignancies including the SOLAR-1 trial in breast cancer (27). The recent TRIUMPH trial (28) provides particularly relevant evidence for the efficacy of PI3Kα inhibition (alpelisib) in squamous cell carcinomas harboring *PIK3CA* mutations, demonstrating a median PFS of 3.4 months in head and neck squamous carcinoma. While these data are derived from a different anatomic site, the shared squamous biology and *PIK3CA*-driven pathogenesis offer translatable insights for PBSCC management.

Similarly, *FGFR* amplification nominates *FGFR* inhibitors as a rational therapeutic strategy, with supportive evidence from trials in *FGFR*-altered solid tumors (29, 30), though the translational relevance to breast cancer requires prospective validation. The convergent molecular features between PBSCC and other squamous carcinomas support the rationale for cross-histology application of targeted therapies, particularly in this orphan disease where prospective trials remain challenging.

### 4.5 Limitations and future directions

The singular nature of this case report necessarily limits the generalizability of our findings. However, the exceptional rarity of HER2-positive PBSCC makes prospective trials challenging. Our experience underscores the value of meticulous documentation and molecular analysis in rare malignancies, where each case contributes meaningfully to understanding disease biology.

Future progress in this area will likely depend on collaborative efforts to establish international registries for rare breast cancer subtypes. Furthermore, basket trials based on molecular alterations rather than tumor origin may offer the most viable path to generating evidence for these orphan populations. Specifically, trials enrolling patients with *PIK3CA*-mutant solid tumors or *FGFR*-amplified squamous carcinomas could provide valuable insights applicable to PBSCC.

In conclusion, our case illustrates how HER2-positive PBSCC employs multiple defensive strategies—from histological barriers and molecular bypass to ultimate antigen loss—to evade targeted therapy. Recognizing this multifaceted resistance nature is essential for developing more effective management strategies for this challenging disease.

## 5 Conclusion

This case of HER2-positive PBSCC demonstrates a multi-driver resistance pattern, where synergistic interaction between *PIK3CA* mutation and *FGFR* amplification led to failure of conventional HER2-targeted therapies. While T-DXd achieved transient response through its topoisomerase I inhibitor payload, treatment ultimately failed due to acquired HER2 antigen loss confirmed by repeat biopsy.

For patients with multi-line HER2-targeted therapy failure and confirmed antigen loss, treatment should shift decisively toward biology-driven precision therapy. PI3Kα inhibitors (e.g., alpelisib) represent the priority choice based on evidence from analogous squamous carcinomas, with *FGFR* inhibitors as a rational secondary option, preferably within clinical trials.

This case validates repeated molecular profiling as essential for identifying resistance mechanisms and supports cross-histology application of targeted therapies for this orphan disease.

## Data Availability

The original contributions presented in the study are included in the article/supplementary material. Further inquiries can be directed to the corresponding author.
